# The Relationship Between Bioelectrical Impedance Analysis Parameters and Laboratory Biomarkers in an Elderly Polish Cohort: A Cross-Sectional Study

**DOI:** 10.3390/nu17243843

**Published:** 2025-12-09

**Authors:** Anna Tomasiewicz, Tomasz Targowski, Sebastian Makuch, Jacek Polański, Wojciech Tański

**Affiliations:** 1 4th Military Clinical Hospital in Wrocław, 50-981 Wroclaw, Poland; anatomasiewicz@gmail.com (A.T.); wojciech.tanski@pwr.edu.pl (W.T.); 2 Department of Geriatrics, National Institute of Geriatrics, Rheumatology and Rehabilitation, 02-637 Warsaw, Poland; tomasz.targowski@spartanska.pl; 3 Department of Clinical and Experimental Pathology, Wrocław Medical University, 50-368 Wroclaw, Poland; s.makuch@umw.edu.pl; 4 Department of Internal and Occupational Diseases, Hypertension and Clinical Oncology, Wrocław Medical University, 50-556 Wroclaw, Poland; 5 Faculty of Medicine, Wrocław University of Science and Technology, 50-376 Wroclaw, Poland

**Keywords:** bioelectrical impedance, laboratory parameters, elderly population, MNA, Leptin

## Abstract

**Background/Objectives:** Assessing age-related health decline in the elderly is critical, yet standard metrics like Body Mass Index (BMI) can be misleading. Bioelectrical impedance analysis (BIA) is a popular method to assess body composition. This study evaluated the relationship between BIA-derived parameters, a comprehensive panel of laboratory biomarkers, and nutritional status in a cohort of Polish older adults. **Methods:** In a cross-sectional study of 126 elderly participants (106 women, 20 men; mean age: 72.4 years), we performed multi-frequency segmental BIA to measure fat-free mass, skeletal muscle mass, and phase angle (PA). Nutritional status was assessed using the Mini Nutritional Assessment (MNA). Venous blood samples were analyzed for a comprehensive panel of hematological, inflammatory (CRP), hormonal (leptin), and metabolic biomarkers. **Results:** The analysis characterized the body composition and biomarker profiles of the cohort. MNA-defined malnutrition risk was associated with significantly lower muscle mass and PA, and altered fluid distribution (higher extracellular-to-total body water ratio), but not with reduced fat mass. Adiposity correlated strongly with leptin and CRP. Hematological parameters were linked to lean mass, while zinc and albumin correlated with PA. Canonical analysis identified two distinct physiological axes: a dominant “adipo-hormonal” axis linking leptin to fat mass, and a secondary “metabolic–cellular integrity” axis linking zinc and iron status to Phase Angle and fluid balance. **Conclusions:** In older adults, nutritional risk is characterized by sarcopenia and fluid shifts, not low adiposity, highlighting the inadequacy of BMI. BIA parameters, especially the phase angle, may serve as promising indicators of cellular health that correlate strongly with key micronutrients, suggesting a potential role in complementary geriatric assessment.

## 1. Introduction

Global population aging reflects a substantial demographic phenomenon that raises serious problems for public health policy and healthcare systems. Malnutrition in this demographic is highly prevalent, affecting approximately 5–10% of community-dwelling elderly and up to 50% of those in rehabilitation or long-term care settings [1]. One of the main issues in geriatric medicine is the effective and early diagnosis of age-related changes, including the insidious onset of sarcopenia (age-related loss of muscle mass and function), malnutrition, and chronic low-grade inflammation. An important component of the overall health in this population is nutritional status, disturbances of which may have far-reaching social and economic consequences, such as more frequent and prolonged hospitalizations, increased healthcare costs, and a decline in quality of life [2,3]. As a result, many methods are being developed to assess nutritional status as accurately as possible, including survey research, body composition assessment, and the use of various laboratory tests. The most commonly used methods for assessing nutritional status are survey-based assessments, including the Mini Nutritional Assessment survey (MNA), as well as anthropometric measurements, the most popular of which are body weight and BMI. However, increasing attention is being paid to the fact that the above methods provide a significantly incomplete assessment. Consequently, the scientific community is seeking to identify ways to supplement this incomplete picture by incorporating selected laboratory investigations and detailed body composition analysis. In routine clinical practice, patient assessment relies heavily on a panel of laboratory biomarkers, including complete blood counts, inflammatory markers like C-reactive protein (CRP), lipid profiles, and various metabolic or hormonal indicators. In this study, we specifically selected leptin and C-reactive protein (CRP) to capture the ‘adipo-inflammatory’ status often observed in aging, while zinc and albumin were chosen as key indicators of protein synthesis and cellular membrane integrity. Among the laboratory markers associated with nutritional status, increasing attention is being paid to parameters reflecting inflammation (such as C-reactive protein—CRP), lipid metabolism (total cholesterol), hormonal profile (leptin, ghrelin), as well as hematological parameters (hemoglobin) and liver enzymes (AST, ALT) [4,5,6]. Diagnostic value is also attributed to markers of micronutrient and protein deficiencies, including zinc and albumin levels [7,8]. Studies indicate that selected biomarkers may correlate not only with nutritional status but also with body composition. For instance, research in Central European populations has demonstrated that metabolic markers such as gamma-glutamyl transferase (GGT) and serum uric acid show significant correlations with fat mass, muscle mass, and body cell mass [9,10,11]. Despite the growing body of evidence linking laboratory parameters to nutritional status, relatively few studies have comprehensively examined how these biomarkers relate to both body composition and self-reported functional limitations in older adults [12]. Moreover, most existing research has either focused on specific clinical subpopulations or assessed nutritional status in isolation, without considering its broader interaction with physiological reserves or subjective health.

Bioelectrical Impedance Analysis (BIA) has emerged as a valuable method for assessing body composition. It is a non-invasive, repeatable, and widely available technique that provides a quantitative evaluation of key physiological parameters, including fat mass, fat-free mass (FFM), and total body water (TBW), along with its distribution into intracellular and extracellular compartments [13]. Beyond these fundamental metrics, BIA yields the phase angle (PA), a parameter derived from the relationship between resistance and reactance of cell membranes. The phase angle is increasingly recognized as a critical indicator of cellular health, reflecting the integrity and functional capacity of cell membranes [14]. However, it is important to note that PA reference values can vary significantly by ethnicity and population specificities; thus population-specific validation is needed. This moves the assessment beyond simple quantification of body compartments toward a qualitative evaluation of tissue health. While traditional anthropometric measures like body mass index (BMI) can be misleading in older adults—for instance in cases of sarcopenic obesity where muscle loss is masked by stable or increased fat mass—BIA offers a more comprehensive view. The phase angle, in particular, provides a deeper physiological insight; a low PA can signal compromised cell membrane integrity and poor muscle quality, even when muscle mass appears adequate, thus serving as a powerful predictor of clinical outcomes, complications, and mortality in geriatric and oncological populations [15].

Despite the utility of the MNA, it relies partially on subjective reporting and traditional anthropometry, which may fail to capture subclinical physiological changes such as cell membrane degradation or early inflammatory fluid shifts. A gap in knowledge remains regarding the extent to which subjective nutritional risk scores correspond to quantifiable cellular and metabolic deficits. Therefore, the primary objective of this study was to evaluate the relationship between selected body composition components (measured by bioelectrical impedance analysis), laboratory biomarkers (CRP, leptin) and nutritional status (MNA). The relationship between micronutrients involved in membrane stability (such as zinc) and BIA-derived Phase Angle remains under-explored in the Central European elderly population. Thus, we hypothesized that BIA-derived cellular health markers, particularly the Phase Angle (PA), would correlate more strongly with specific nutritional biomarkers such as zinc and albumin, than traditional anthropometric measures like BMI.

## 2. Materials and Methods

### 2.1. Study Design

This cross-sectional study investigated a cohort of older adults residing in Wrocław, Poland. Participants were recruited from community centers (Universities of the Third Age) and nursing homes under the municipal ‘Złote lata w zdrowiu’ health program. The inclusion criteria for the study were an age of 58 years or older and the ability to provide written informed consent. Individuals were excluded if they had implanted electrical devices such as a cardiac pacemaker, presented with generalized edema, or had acute or unstable chronic conditions that precluded the safe performance of measurements.

The final study cohort consisted of 126 participants, comprising 106 women (84.1%) and 20 men (15.9%). The age of the participants ranged from 58 to 92 years, with a mean age of 72.4 ± 5.6 years. This sex distribution reflects the demographic structure of the older population in the region. All study procedures were conducted in accordance with the ethical principles of the Declaration of Helsinki. Written informed consent was obtained from every participant prior to their inclusion.

### 2.2. Data Collection Procedures

Sociodemographic and clinical data were gathered using standardized questionnaires and a review of available medical documentation. Information collected included age, sex, level of education, marital status, and current housing situation. A detailed medical history was obtained to document the prevalence of chronic diseases.

Nutritional status was formally assessed using the validated Polish version of the Mini Nutritional Assessment (MNA) scale. The MNA is a comprehensive tool that incorporates anthropometric measurements, dietary history, and functional assessment to screen for malnutrition. Based on their scores, participants were categorized as having a ‘Proper nutritional status’ or being at ‘Risk of malnutrition’.

### 2.3. Bioelectrical Impedance Analysis (BIA)

Whole-body impedance measurements were performed in the morning following an overnight fast of at least 8 h and abstinence from excessive fluid intake. Participants were instructed to abstain from alcohol consumption for 24 h and from vigorous physical activity for 12 h prior to the assessment. All subjects were asked to void their bladders within 30 min of the measurement.

Measurements were conducted at the same time of day for all participants. Subjects were assessed in a standing position following a 5–10 min rest period, while barefoot and wearing light clothing, with the upper limbs slightly abducted and the legs separated.

Bioelectrical impedance analysis was performed using a SECA mBCA 555 device (Hamburg, Germany), in accordance with the manufacturer’s instructions. The device was calibrated regularly following the manufacturer’s recommendations.

Exclusion criteria for the BIA included pregnancy, the presence of implanted cardiac devices (pacemakers, ICDs), and clinical signs of severe fluid and electrolyte disturbances.

Body composition was evaluated using a medical-grade, multi-frequency segmental BIA analyzer. Measurements were performed using low-amperage electrical currents at frequencies of 5, 7.5, 50, and 75 kHz. To ensure the accuracy and reproducibility of the measurements, a strict protocol was followed. Participants were instructed to be in a fasted state for a minimum of 8 h and to refrain from physical activity prior to the assessment. All measurements were conducted under standardized environmental conditions with the participant in a resting state. The BIA device underwent regular calibration and quality control checks as per the manufacturer’s recommendations.

The following key parameters were recorded for each participant: Fat-Free Mass (FFM), Skeletal Muscle Mass (SMM), Total Body Water (TBW), Extracellular Water (ECW), the ratio of extracellular to total body water (ECW/TBW), and Phase Angle (PA). Additionally, segmental values for resistance (R) and reactance (Xc) were obtained for the trunk, right and left arms, and right and left legs.

### 2.4. Laboratory Analyses

Venous blood samples were collected from all participants in the morning following an overnight fast. All biochemical analyses were performed at a certified medical laboratory operating under PN-EN ISO 15189 [16] quality assurance standards. The comprehensive panel of laboratory tests included:

- Hematology: Hemoglobin (HGB, g/dL), Hematocrit (HCT, %), Red Blood Cell count (RCB, 10^6^/μL), Mean Corpuscular Volume (MCV, fL), Mean Corpuscular Hemoglobin (MCH, pg), and Iron (Fe, μg/dL).
- Inflammation: High-sensitivity C-reactive protein (CRP, mg/L).
- Hormones and Metabolites: Leptin (ng/mL), Ghrelin (ng/mL), 25-hydroxyvitamin D (25(OH)D, ng/mL), and Zinc (Zn, μmol/L).
- Biochemistry and Lipids: Albumin (g/dL), Calcium (Ca, mmol/L), Total Cholesterol (mg/dL), High-Density Lipoprotein (HDL, mg/dL), Low-Density Lipoprotein (LDL-D, mg/dL), and Triglycerides (TG, mg/dL).

### 2.5. Statistical Analysis

For quantitative variables, basic descriptive statistics were calculated: means (M), standard deviations (SD), minimum (Min) and maximum (Max) values, medians (Me), and lower (Q1) and upper (Q3) quartiles. Qualitative (nominal and ordinal) variables were presented in contingency tables as frequencies (*n*) and percentages (%). The Shapiro–Wilk test was used to confirm that the empirical distributions of quantitative variables did not significantly deviate from a normal distribution. For normally distributed quantitative data, the significance of differences in means between two groups was verified using the t-test; when the data did not meet the assumptions of normality, the Mann–Whitney U test was used. For a larger number of groups (e.g., three levels of frailty), the Kruskal–Wallis test was used, followed by Dunn’s test as a post hoc test. In 2 × 2 contingency tables, Fisher’s exact test was used to verify the independence of variables, whereas in 2 × 3 tables, Pearson’s chi-squared test with Yates’s correction was used.

Univariate and multivariate logistic regression analysis was used to estimate the probability of malnutrition risk. Model parameters were estimated using the maximum likelihood method, and their statistical significance was checked with the Wald test. The Box–Tidwell test was used to verify the linear relationship of the logit transformation with continuous independent variables. For statistically significant predictors of malnutrition, odds ratios (ORs) and their 95% confidence intervals (CIs) were estimated.

For continuous variables, Receiver Operating Characteristic (ROC) curve analysis and Youden’s index values were used to determine the cut-off values for predicting the risk of malnutrition. For each predictor, the area under the curve (AUC) was calculated, and for the cut-off values, sensitivity and specificity were estimated. The strength and significance of the correlation between two quantitative variables were assessed by calculating Spearman’s rank correlation coefficient (rho). Canonical analysis was selected to analyze the relationship between two sets of variables (the biochemical profile vs. the body composition profile), rather than analyzing single predictors in isolation as in standard regression models. Given the exploratory nature of this study, a strict Bonferroni correction for multiple comparisons was not applied to avoid increasing Type II error rates; instead, interpretation focused on effect sizes and clinical consistency. For Canonical Correlation Analysis, the sample size (*n* = 126) satisfied the recommended minimum ratio of 10 observations per variable, ensuring adequate statistical power to detect multivariate relationships. Furthermore, a post hoc power analysis indicated that the sample size provided >80% power to detect correlation coefficients of r > 0.25 at a significance level of α = 0.05, which is sufficient for the exploratory nature of this study.

**Figure 1:**
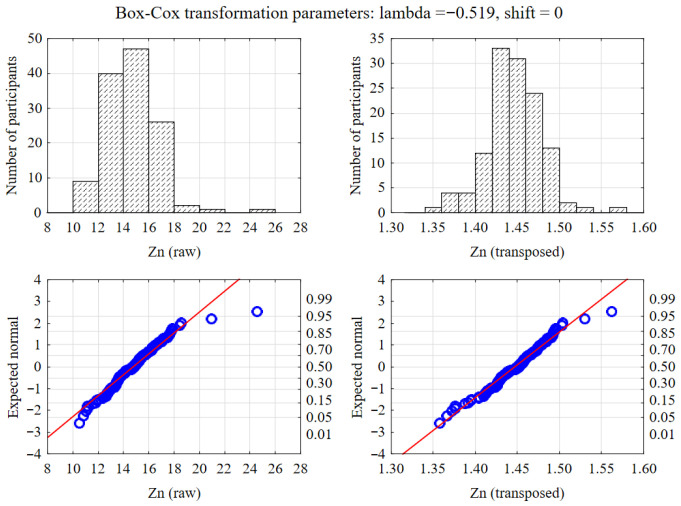
Normalization of data distribution using Box–Cox transformation. The upper panels display histograms of raw (**left**) and transformed (**right**) zinc levels, while the lower panels show the corresponding Q-Q normality plots.

Previously, variables whose distribution deviated from normal were subjected to a Box–Cox transformation. As illustrated in Figure 1, this method effectively normalized the distribution of variables such as Zinc. All statistical analyses were conducted at a significance level of α = 0.05, and on the graphs, variables were presented as medians and interquartile ranges, and performed using Statistica software v.13.3 (TIBCO Software Inc., Palo Alto, CA, USA).

## 3. Results

### 3.1. Participant Characteristics

The final study cohort comprised 126 participants, predominantly women (*n* = 106, 84.1%) with a smaller contingent of men (*n* = 20, 15.9%). The mean age of the cohort was 72.4 ± 5.6 years, with an age range of 58 to 92 years. No statistically significant difference in age was observed between female and male participants (*p* = 0.659).

Sociodemographic characteristics revealed significant differences in living arrangements between the sexes. Men were significantly more likely than women to be partnered (80.0% vs. 32.1%; *p* < 0.001) and to live with a spouse (75.0% vs. 28.3%; *p* < 0.001). Conversely, women were more likely to be widowed (38.7% vs. 15.0%) and to live alone (59.4% vs. 20.0%).

Nutritional status, as assessed by the Mini Nutritional Assessment (MNA), indicated that the cohort was generally well nourished, with a median MNA score of 25.5 (Interquartile Range: 24 to 27). While a trend towards better nutritional status was observed in men (Median MNA: 27) compared to women (Median MNA: 25.5), this difference did not reach the conventional threshold for statistical significance (*p* = 0.05).

The most frequently reported chronic diseases in the cohort were arterial hypertension (43.6%), hyperlipidemia (31.8%), and gastroesophageal reflux disease (29.4%). A statistically significant sex-based difference was found only for gastroesophageal reflux disease, which was substantially more prevalent in women than in men (34.0% vs. 5.0%; *p* = 0.007).

### 3.2. Baseline Characteristics and Body Composition Profile

Bioimpedance and laboratory parameters are presented in Table 1, Table 2 and Table 3, stratified by sex to provide descriptive context for the cohort.

#### 3.2.1. Anthropometry and Body Composition

As detailed in Table 1 and Table 2, men were, on average, significantly taller (1.75 ± 0.06 m vs. 1.59 ± 0.06 m; *p* < 0.001), heavier (84.4 ± 11.9 kg vs. 68.6 ± 13.1 kg; *p* < 0.001), and had a larger waist circumference (1.00 ± 0.11 m vs. 0.90 ± 0.12 m; *p* = 0.001) than women. Despite these differences in body size, Body Mass Index (BMI) was not significantly different between the sexes (27.6 ± 3.3 kg/m^2^ for men vs. 27.1 ± 5.0 kg/m^2^ for women; *p* = 0.55).

In contrast to BMI, direct measures of body composition showed highly significant differences. Women exhibited a significantly greater relative fat mass (41.5% ± 5.3% vs. 31.4% ± 6.7%; *p* < 0.001) and a higher Fat Mass Index (FMI) (11.42 ± 3.33 kg/m^2^ vs. 8.81 ± 2.77 kg/m^2^; *p* = 0.001). Conversely, men demonstrated substantially greater absolute fat-free mass (FFM) (57.5 ± 6.5 kg vs. 39.7 ± 5.9 kg; *p* < 0.001) and skeletal muscle mass (SMM) (27.1 ± 4.0 kg vs. 17.1 ± 3.5 kg; *p* < 0.001, Cohen’s d = 2.79). This superiority in muscle mass for men was consistent across all measured body segments, including the trunk (SMM TO), right and left legs (SMM RL, SMM LL), and right and left arms (SMM RA, SMM LA) (*p* < 0.001 for all). Consequently, men had a significantly higher Fat-Free Mass Index (FFMI) (18.8 ± 1.5 kg/m^2^ vs. 15.6 ± 2.0 kg/m^2^; *p* < 0.001).

These differences in lean mass were reflected in body water compartments and electrical properties. Men had significantly greater volumes of total body water (TBW) and extracellular water (ECW) (*p* < 0.001 for both). Consistent with their greater muscle and water content, which facilitates electrical current flow, men displayed significantly lower electrical resistance (R) and, at higher frequencies, higher reactance (Xc) across multiple body segments and frequencies (Table S1). These fundamental biophysical differences culminated in men having a significantly higher mean Phase Angle (PA), an indicator of cellular health and integrity, compared to women (4.89° ± 0.57° vs. 4.42° ± 0.51°; *p* < 0.001, Cohen’s d = 0.90)

#### 3.2.2. Laboratory Biomarkers

Statistically significant sex-based differences were also evident in the panel of laboratory biomarkers (Table 3). Men presented with significantly higher concentrations of iron (Fe) (*p* = 0.016), hemoglobin (HGB) (*p* = 0.002), hematocrit (HCT) (*p* = 0.009), and mean corpuscular hemoglobin (MCH) (*p* = 0.021). In the white blood cell differential, men had higher monocyte counts (*p* = 0.015). In contrast, women had significantly higher levels of High-Density Lipoprotein (HDL) cholesterol (*p* < 0.001) and lower platelet counts (PLT) (*p* = 0.004). Additionally, women exhibited significantly lower serum concentrations of 25-hydroxyvitamin D (39.9 ± 14.6 ng/mL vs. 33.2 ± 10.2 ng/mL; *p* = 0.017).

### 3.3. Association of MNA-Defined Nutritional Status with Body Composition

To investigate the relationship between a validated nutritional screening tool and objective body composition measures, participants were stratified into two groups based on their MNA score: ‘Proper nutritional status’ (*n* = 97) and ‘Risk of malnutrition’ (*n* = 29). The comparison of BIA parameters between these groups is presented in Table 4.

Participants at risk of malnutrition were characterized by significantly lower body weight (Median: 65.2 kg vs. 71.0 kg; *p* = 0.004) and height (Median: 1.58 m vs. 1.62 m; *p* = 0.018). This corresponded to lower energy requirements, with significantly reduced resting energy expenditure (REE) (*p* = 0.002) and total energy expenditure (TEE) (*p* = 0.034) in the at-risk group.

The most striking differences were observed in measures of lean body mass and hydration. The group at risk of malnutrition demonstrated significantly lower FFM (Median: 38.3 kg vs. 42.3 kg; *p* = 0.001), SMM (Median: 15.8 kg vs. 18.7 kg; *p* < 0.001, Cohen’s d = 0.74), and FFMI (Median: 15.4 kg/m^2^ vs. 16.2 kg/m^2^; *p* = 0.003). This deficit in muscle mass was systemic, with significantly lower values observed in all segmental SMM measurements (*p* ≤ 0.001). Correspondingly, total body water was also significantly lower in the at-risk group (*p* = 0.001).

In contrast, no significant differences were found in parameters related to adiposity. Measures of relative fat mass (*p* = 0.467), absolute fat mass (*p* = 0.138), and FMI (*p* = 0.616) were comparable between the two nutritional status groups.

Parameters reflecting cellular health and fluid balance were also significantly different. The at-risk group had a significantly lower Phase Angle (Median: 4.36° vs. 4.44°; *p* = 0.016, Cohen’s d = 0.15). Furthermore, this group exhibited a significantly higher ratio of extracellular to total body water (ECW/TBW) (Median: 49.9% vs. 48.2%; *p* = 0.001, Cohen’s d = 0.71), indicating a relative expansion of the extracellular fluid compartment.

### 3.4. Bivariate Correlations Between BIA Parameters and Laboratory Biomarkers

Spearman’s rank correlation analysis was performed to elucidate the relationships between individual BIA parameters and laboratory biomarkers across the entire cohort. The results are summarized in Table 4, Table S2, Table S3, Table S4 and Table S5. The correlation coefficient values between selected BIA parameters and laboratory test results have been shown in Table 5.

**Figure 2:**
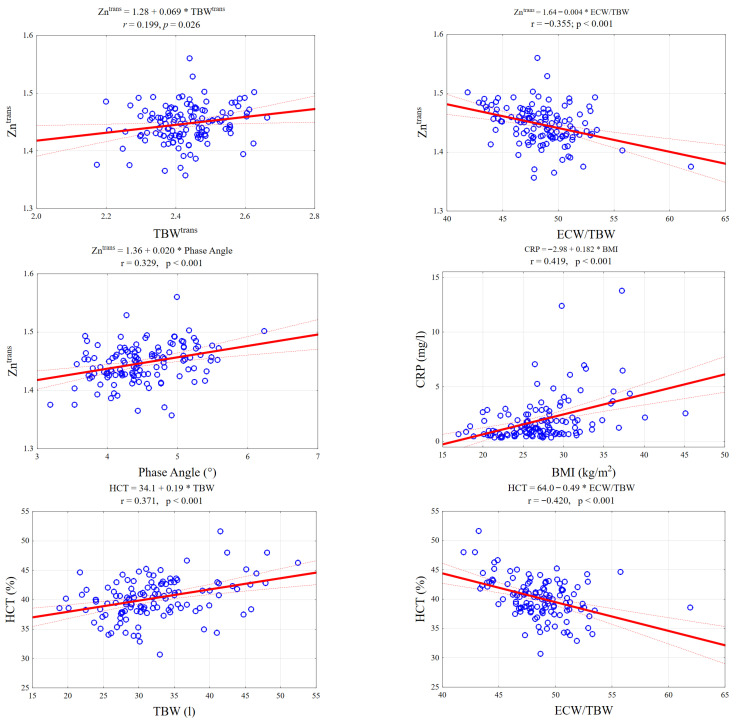
Scatterplots illustrating key significant correlations between BIA parameters and laboratory biomarkers. The panels display linear regression fits (solid red line) with 95% confidence intervals (dotted red lines) for selected pairs: (**Top row**) Zinc levels positively correlate with Total Body Water (TBW) and negatively with the ECW/TBW ratio. (**Middle row**): Zinc shows a significant positive association with Phase Angle; CRP levels rise with increasing BMI. (**Bottom row**): Hematocrit (HCT) is positively linked to TBW but inversely related to the ECW/TBW ratio, reflecting fluid distribution shifts.

As shown in Figure 2, specific laboratory markers demonstrated distinct linear relationships with BIA parameters. Notably, serum zinc levels exhibited a positive correlation with Total Body Water and Phase Angle, while showing a negative association with the ECW/TBW ratio. Conversely, inflammatory markers like CRP increased linearly with BMI, reinforcing the link between adiposity and inflammation.

#### 3.4.1. Adiposity, Inflammation, and Hormonal Status

Measures of adiposity were strongly associated with the hormone leptin and the inflammatory marker C-reactive protein (CRP). Leptin showed the most powerful correlations, with very strong positive associations with FMI (*r_s_* = 0.763), absolute fat mass (*r_s_* = 0.727), relative fat mass (*r_s_* = 0.666), and BMI (*r_s_* = 0.660). CRP was also moderately and positively correlated with these same measures of adiposity: BMI (*r_s_* = 0.429), absolute fat mass (*r_s_* = 0.439), and FMI (*r_s_* = 0.426). A significant positive correlation was also found between CRP and visceral adipose tissue (VAT) (*r_s_* = 0.281).

#### 3.4.2. Lean Mass, Hydration, and Hematological Status

Hematological parameters reflecting red blood cell mass were significantly associated with lean body mass. Hemoglobin (HGB) and hematocrit (HCT) were positively correlated with both FFM (*r_s_* = 0.400 and *r_s_* = 0.359, respectively) and SMM (*r_s_* = 0.432 and *r_s_* = 0.391, respectively). A particularly strong and significant negative correlation was observed between these hematological markers and the ECW/TBW ratio (HGB: *r_s_* = −0.465; HCT: *r_s_* = −0.392), indicating that lower red cell mass is associated with a relative increase in extracellular fluid.

#### 3.4.3. Micronutrient Status and Cellular Health

Key micronutrients and proteins were associated with markers of cellular health and fluid balance. Serum zinc (Zn) concentration was positively correlated with SMM (*r_s_* = 0.223) and showed a strong positive correlation with Phase Angle (*r_s_* = 0.321). Zinc was also strongly and negatively correlated with the ECW/TBW ratio (*r_s_* = −0.340). Similarly, serum albumin was positively correlated with Phase Angle (*r_s_* = 0.271) and negatively correlated with the ECW/TBW ratio (*r_s_* = −0.218). In contrast, 25-hydroxyvitamin D levels were weakly but significantly negatively correlated with FFM (*r_s_* = −0.202) and SMM (*r_s_* = −0.206).

#### 3.4.4. Phase Angle as an Integrative Marker

Phase Angle (PA) demonstrated significant positive correlations with a range of biomarkers indicative of favorable health status. This included hematological parameters (HGB: *r_s_* = 0.360; HCT: *r_s_* = 0.317), markers of protein and micronutrient status (albumin: *r_s_* = 0.271; zinc: *r_s_* = 0.321), and metabolic markers such as alanine aminotransferase (ALT) (*r_s_* = 0.277) and triglycerides (TG) (*r_s_* = 0.238).

### 3.5. Multivariate Relationship Between Body Composition and Laboratory Profiles

To explore the complex, multidimensional relationships between the set of BIA variables and the set of laboratory variables (LAB), canonical correlation analysis was performed. The analysis revealed a robust and highly significant overall relationship between the two sets of variables. The strongest canonical correlation was R = 0.801 (*p* < 0.000001), indicating a substantial shared variance between the domains of body composition and biochemistry.

The first, and most dominant, canonical variate described a powerful relationship primarily driven by adiposity. Within the laboratory variable set, transformed leptin had the highest canonical weight (1.01) and factor loading (0.978). Within the BIA variable set, relative fat mass (RFM) had the highest corresponding weight (1.12). This variate clearly represents a strong “adipo-hormonal” axis, linking the primary hormonal signal of fat stores (leptin) with the direct BIA measurement of body fatness.

The second significant canonical variate captured an independent relationship reflecting metabolic and cellular status. The primary contributors from the laboratory set were iron (Fe) (weight = 0.56) and transformed zinc (Zn) (weight = 0.55). These were most strongly related to a combination of BIA variables, including Phase Angle (weight = 1.41), ECW/TBW ratio (weight = 0.96), and total energy expenditure (TEE) (weight = 0.62). This second variate can be interpreted as a “metabolic–cellular integrity” axis, linking micronutrient status (iron and zinc) to BIA-derived measures of cellular health (PA), hydration status (ECW/TBW), and overall metabolic rate (TEE).

For the LAB set, for the first canonical variable, the variable LEPTIN shows a very high factor loading (0.978) (Table S6). This variable explains 14.7% of the variance in this dataset. For the second canonical variable, the variables Fe (0.518) and Zn (0.649) have high factor loadings. This confirms the decisive influence of these variables. For the right set (BIA) and the first canonical variable, the variables BMI and RFM contribute the largest factor loading. And for the second canonical variable, the variables TEE, PA, and ECW/TBW have the largest loading.

A redundancy analysis showed that the total redundancy was 18.4% (Table S7). A redundancy value of 18.4% means that less than one-fifth of the variance in one set of variables is explained by the second set of variables. This result indicates the presence of a certain, but not very strong, common area of variability between the LAB and BIA sets. In the context of the research conclusions, this would mean that the relationships between the variables are present but not dominant, and there are many other factors influencing the variance that were not captured by the other set of variables.

## 4. Discussion

This study provides a comprehensive analysis of the intricate relationships between body composition, assessed by multi-frequency segmental bioelectrical impedance, and a broad panel of laboratory biomarkers in a cohort of Polish older adults. First of all, the study demonstrates that nutritional risk, as identified by the MNA, is strongly characterized by deficits in lean and skeletal muscle mass and altered fluid distribution, rather than by a simple reduction in adiposity, pointing towards a phenotype of sarcopenic risk. Moreover, the analysis reveals that specific laboratory biomarkers are linked to distinct body composition domains: leptin and CRP are associated with adiposity and its inflammatory state; hematological parameters are linked to sarcopenia and fluid shifts; and zinc and albumin are correlated with cellular integrity. Also, the BIA-derived phase angle has been shown as a potent, integrative indicator of overall health, correlating with markers of muscle mass, protein status, and micronutrient sufficiency. The canonical correlation analysis demonstrated two primary physiological axes: a dominant “adipo-hormonal” axis and a secondary “metabolic–cellular integrity” axis, providing a novel view of the interplay between biochemistry and body composition in aging. One of the most critical clinical implications of this finding arises from the observation that most differences in body composition occurred in the absence of a statistically significant difference in BMI between the sexes (*p* = 0.55). This discrepancy exposes the severe limitations of relying on BMI as a primary tool for nutritional and health assessment in older adults, which is in line with other studies [17,18].

The Mini Nutritional Assessment (MNA) is a widely accepted and validated screening tool for identifying older adults at risk of malnutrition [19]. A key strength of the present study is the objective validation of MNA-defined nutritional risk using BIA. Our results show that participants classified as being at ‘Risk of malnutrition’ had significantly lower FFM, SMM, and phase angle, coupled with a significantly higher ECW/TBW ratio. These findings provide a clear physiological signature for a positive MNA screen, linking it directly to quantifiable deficits in high-quality functional tissue (muscle) and an expansion of the extracellular fluid space, which are hallmarks of sarcopenia, cachexia, and compromised cellular integrity. This BIA profile aligns perfectly with recent research linking these parameters to distinct body composition domains. A lower PhA is a strong correlate of reduced skeletal muscle and cellular integrity, while an elevated ECW/TBW ratio reflects increased adiposity and systemic fluid imbalance [20]. Therefore, our findings provide direct evidence that a positive MNA screen identifies a phenotype of sarcopenia and compromised cellular health, rather than simple energy deficiency. Furthermore, the clinical significance of this BIA signature is underscored by evidence linking it directly to functional outcomes. The specific combination of a lower PhA and an elevated ECW/TBW ratio has been identified as a powerful, independent predictor of poor muscle function, including low grip strength and physical performance in older adults [21]. By connecting the MNA score to this high-risk BIA profile, our study bridges the gap between a simple screening tool and a quantifiable state of sarcopenia that precedes functional decline.

The strong, positive correlations observed between CRP and all measures of adiposity, including visceral adipose tissue, provide direct evidence in our cohort for the concept of “inflammaging”—a state of chronic, low-grade inflammation driven by metabolically active adipose tissue [22]. While this adipose tissue-inflammation link is established in other populations, our study represents one of the first comprehensive integrations of multi-frequency BIA with this specific biomarker panel in a Central European elderly cohort, providing regional validation of these physiological patterns. Our finding is not unique to the elderly but appears to be a fundamental biological process that persists across the lifespan. A recent meta-analysis in over 4600 adolescents confirmed this same positive correlation between body fat percentage, CRP, and leptin, establishing this adipo-inflammatory link early in life [23]. This is further supported by our own observation of an extremely strong correlation between serum leptin and fat mass, consistent with leptin’s role as a key adipokine This relationship reflects the endocrine function of adipose tissue. Hypertrophic adipocytes secrete pro-inflammatory adipokines—including leptin and cytokines such as TNF-α and IL-6—which stimulate hepatic CRP production [24]. This state of chronic low-grade inflammation promotes protein catabolism and oxidative stress, directly compromising cell membrane stability. Biophysically, this loss of membrane integrity and the accompanying fluid shifts are captured by BIA as a reduced Phase Angle and an elevated ECW/TBW ratio, linking excess adiposity directly to cellular decline.

Perhaps the most novel finding in this domain is the strong inverse correlation between HGB/HCT and the ECW/TBW ratio (*r_s_* = −0.465 for HGB). This result is not isolated to our cohort; a recent study in 371 patients with diabetes independently confirmed that a high ECW/TBW ratio is a significant and independent predictor of lower hemoglobin and hematocrit levels [25]. This relationship highlights that a lower red blood cell mass is associated with a relative expansion of the extracellular fluid compartment. This link can be explained by two primary mechanisms. First, a high ECW/TBW ratio can cause relative hemodilution, directly lowering the concentration of red blood cells. Such fluid overload is often a sign of underlying cardiac or renal dysfunction, conditions also known to drive muscle wasting. Second, shared pathophysiology, such as systemic inflammation or severe protein-energy malnutrition, can simultaneously suppress red blood cell production while also causing fluid shifts due to reduced oncotic pressure.

Moreover, our findings strongly corroborate the clinical value of phase angle (PA) as a global marker of health. The significantly lower PA in our MNA-defined at-risk group aligns with research demonstrating that low PA is a common feature in populations with metabolic syndrome, where higher PA is consistently linked to better body composition, specifically greater muscle mass [26]. This reinforces our observation that PA positively correlated with markers of robust physiological status like albumin and hemoglobin.

The link between PA and zinc (*r_s_* = 0.321) is particularly insightful. Zinc serves as an essential stabilizer of cell membrane structure and a cofactor for antioxidant enzymes like superoxide dismutase. Since Phase Angle directly reflects membrane capacitance and integrity, a zinc deficiency likely exacerbates oxidative stress-induced membrane damage, manifesting as a lower Phase Angle. Similarly, albumin acts as the major extracellular antioxidant; its correlation with PA suggests that compromised protein status parallels cellular vulnerability to oxidative damage. Studies in clinical populations, such as incident hemodialysis patients, show that serum zinc itself is associated with better nutritional and fluid volume markers, reflecting the same state of cellular health that PA captures [27]. A zinc deficiency can impair cellular function, which would be directly reflected as a lower PA.

These convergent relationships position PA not as just another BIA parameter, but as a composite, non-invasive biomarker—a “check engine light” for geriatricians. It integrates information on nutritional adequacy and cellular function, signaling a need for further investigation. From a statistical perspective, the difference in Phase Angle between nutritional groups was significant (*p* = 0.016). However, the absolute difference was small (approx. 0.1°), resulting in a modest effect size (Cohen’s d = 0.15). Therefore, while low PA is a valid population-level signal of nutritional risk, its clinical utility for individual diagnosis should be interpreted with caution and always in combination with other clinical metrics.

This study possesses several notable strengths. Its primary strength is the comprehensive dataset integrating multi-frequency, segmental BIA with an extensive panel of biomarkers, which is uncommon in studies of older adults. The use of advanced statistical techniques, specifically canonical correlation analysis, allowed for an exploration beyond simple pairwise correlations. Furthermore, including participants from both community and institutional settings enhances the generalizability of the findings.

Nevertheless, the study has limitations. The cross-sectional design precludes any inference of causality, such as the temporal relationship between low zinc and phase angle. Furthermore, an a priori sample size calculation was not performed for this exploratory study. While the sample was sufficient to detect moderate-to-strong correlations, the study may lack statistical power to identify smaller effect sizes. A significant sex imbalance (84.1% female) limits the statistical power for robust sex-specific analyses and tempers generalizability to elderly men. Consequently, the findings predominantly reflect the physiology of older women, and caution should be exercised when generalizing these results to the male elderly population. Moreover, we did not quantify diet or physical activity, nor did we control for hydration-altering medications (diuretics, antihypertensives), which may limit the precision of BIA-derived fluid indices. Finally, the dataset contained acknowledged data anomalies, including outliers and a data entry error, though their influence was mitigated by the use of non-parametric methods.

## 5. Conclusions

Nutritional risk among older adults is often characterized by a sarcopenic and fluid-imbalanced phenotype, which is frequently undetectable using BMI alone. The analysis revealed a strong correlation between inflammation and body fat, as well as a novel metabolic–cellular integrity axis that links micronutrient status, specifically zinc and iron, to cell membrane health, as measured by Phase Angle. These results support the routine incorporation of Phase Angle and extracellular water-to-total body water (ECW/TBW) ratios into geriatric assessments as non-invasive markers of cellular decline that enhance the effectiveness of screening tools.

## Figures and Tables

**Table 1:** Results of bioelectrical impedance analysis in a group of 126 subjects, divided by sex.

Bioimpedance Parameters Mean ± *SD*	Total *n* = 126	Women *n* = 106	Men *n* = 20	*p*-Value
BMI (kg/m^2^)	27.1 ± 4.7	27.1 ± 5.0	27.6 ± 3.3	0.55
Relative fat mass (%)	39.9 ± 6.7	41.5 ± 5.3	31.4 ± 6.7	<0.001
Absolute fat mass (kg)	28.6 ± 8.4	28.9 ± 8.5	26.9 ± 8.4	0.34
Fat-free mass FFM (kg)	42.5 ± 8.9	39.7 ± 5.9	57.5 ± 6.5	<0.001
Skeletal muscle mass (kg)	18.7 ± 5.1	17.1 ± 3.5	27.1 ± 4.0	<0.001
SMM TO (kg)	8.3 ± 2.5	7.5 ± 1.6	12.7 ± 1.8	<0.001
SMM RL (kg)	4.2 ± 1.0	3.9 ± 0.9	5.5 ± 0.9	<0.001
SMM LL (kg)	4.2 ± 1.1	3.9 ± 0.9	5.6 ± 1.0	<0.001
SMM LA (kg)	1.0 ± 0.4	0.9 ± 0.2	1.6 ± 0.3	<0.001
SMM RA (kg)	1.0 ± 0.4	0.9 ± 0.2	1.7 ± 0.3	<0.001
Total Body Water (L)	30.0 ± 4.5	42.5 ± 4.5	32.0 ± 4.9	<0.001
Extracellular Water (L)	15.4 ± 2.6	14.7 ± 2.1	19.1 ± 1.9	<0.001

BMI: Body Mass Index; FFM: Fat-Free Mass; SMM: Skeletal Muscle Mass; TO: Trunk; RL: Right Leg; LL: Left Leg; LA: Left Arm; RA: Right Arm; TBW: Total Body Water; ECW: Extracellular Water; R: Resistance; Xc: Reactance; LB: Left Body; RB: Right Body; TEE: Total Energy Expenditure; REE: Resting Energy Expenditure; FFMI: Fat-Free Mass Index; FMI: Fat Mass Index; PA: Phase Angle.

**Table 2:** Results of bioelectrical impedance analysis in a group of 126 people divided by sex.

Bioimpedance Parameters Mean ± *SD*	Total *n* = 126	Women *n* = 106	Men *n* = 20	*p*-Value
Waist circumference (m)	0.92 ± 0.13	0.90 ± 0.12	1.00 ± 0.11	0.001
Weight (kg)	71.1 ± 14.2	68.6 ± 13.1	84.4 ± 11.9	<0.001
Height (cm)	1.62 ± 0.08	1.59 ± 0.06	1.75 ± 0.06	<0.001
TEE (kcal)	2304 ± 425	2194 ± 359	2880 ± 249	<0.001
REE (kcal)	1351 ± 231	1287 ± 183	1687 ± 153	<0.001
Energy Content (×10^−3^ kcal)	317.6 ± 83.6	317.4 ± 84.3	318.7 ± 81.2	0.950
FFMI (kg/m^2^)	16.1 ± 2.2	15.6 ± 2.0	18.8 ± 1.5	<0.001
FMI (kg/m^2^)	11.01 ± 3.38	11.42 ± 3.33	8.81 ± 2.77	0.001
Z (FFMI)	−0.55 ± 1.41	−0.54 ± 1.48	−0.59 ± 0.99	0.879
Z (FMI)	0.94 ± 1.02	0.94 ± 1.03	0.89 ± 0.98	0.834
Phase Angle (°)	4.49 ± 0.54	4.42 ± 0.51	4.89 ± 0.57	<0.001
VAT	12.9 ± 2.0	13.0 ± 1.9	12.8 ± 2.5	0.712
ECW/TBW (%)	13.7 ± 2.5	13.8 ± 2.5	13.1 ± 2.5	0.264

TEE: Total Energy Expenditure; REE: Resting Energy Expenditure; EC: Energy Content; FFMI: Fat-Free Mass Index; FMI: Fat Mass Index; Z (FFMI): Z-score (standardized score) for Fat-Free Mass Index; Z (FMI): Z-score (standardized score) for Fat Mass Index; ECW/TBW: Extracellular Water-to-Total Body Water ratio; VAT: Visceral Adipose Tissue.

**Table 3:** Laboratory test results in a group of 126 people, divided by sex.

Laboratory Test Results Mean ± *SD*	Total *n* = 126	Women *n* = 106	Men *n* = 20	*p*-Value
Ghrelin (ng/mL)	1.77 ± 1.11	1.78 ± 1.10	1.72 ± 1.20	0.828
Leptin (ng/mL)	8.1 ± 14.3	8.9 ± 14.7	3.7 ± 11.0	0.078
Fe (μg/dL)	98.4 ± 30.6	95.4 ± 29.8	114.4 ± 30.3	0.016
CRP (mg/L)	1.96 ± 2.06	1.89 ± 1.81	2.36 ± 3.07	0.515
Ca (mmol/L)	2.36 ± 0.10	2.36 ± 0.10	2.36 ± 0.09	0.829
Albumins (g/dL)	4.29 ± 0.22	4.28 ± 0.22	4.34 ± 0.21	0.299
Zinc (μmol/L)	15.0 ± 3.5	14.9 ± 3.8	15.6 ± 1.7	0.164
Vitamin D (ng/mL)	38.9 ± 14.2	39.9 ± 14.6	33.2 ± 10.2	0.017
Total cholesterol (mg/dL)	195 ± 43	198 ± 43	181 ± 41	0.126
HDL (mg/dL)	59.4 ± 13.8	61.4 ± 13.5	47.6 ± 8.7	<0.001
Non-cholesterol (mg/dL)	136 ± 40	136 ± 41	133 ± 36	0.771
LDL-D (mg/dL)	117 ± 39	118 ± 39	111 ± 37	0.520
TG (mg/dL)	106 ± 46	102 ± 44	124 ± 52	0.119
AST (IU/L)	26.6 ± 8.2	26.2 ± 7.6	29.3 ± 10.8	0.235
ALT (IU/L)	23.3 ± 14.4	22.6 ± 14.5	27.4 ± 13.6	0.170
HGB (g/dL)	13.2 ± 1.5	13.0 ± 1.3	14.4 ± 1.6	0.002
HCT (%)	40.2 ± 3.3	39.7 ± 2.8	42.9 ± 4.6	0.009
RCB (×10^6^/μL)	4.76 ± 4.00	4.78 ± 4.34	4.67 ± 0.62	0.807
MCV (fL)	91.6 ± 4.2	91.4 ± 3.7	92.5 ± 6.3	0.466
MCH (pg)	30.3 ± 1.6	30.1 ± 1.4	31.0 ± 2.0	0.021
MCHC (g/dL)	35.6 ± 27.8	35.9 ± 30.3	33.6 ± 0.7	0.426
RDW-CV (%)	13.3 ± 0.7	13.3 ± 0.7	13.1 ± 0.6	0.414
WBC (×10^3^/μL)	6.09 ± 1.55	6.01 ± 1.50	6.52 ± 1.82	0.263
LYMPH (×10^3^/μL)	1.99 ± 1.05	2.01 ± 1.12	1.86 ± 0.61	0.408
MONO (×10^3^/μL)	0.51 ± 0.16	0.48 ± 0.14	0.62 ± 0.21	0.015
NEUT (×10^3^/μL)	3.42 ± 1.10	3.36 ± 1.01	3.77 ± 1.48	0.260
EOS (×10^3^/μL)	0.18 ± 0.12	0.18 ± 0.12	0.18 ± 0.14	0.783
BASO (×10^3^/μL)	0.04 ± 0.02	0.04 ± 0.02	0.04 ± 0.02	0.936
IG (×10^3^/μL)	0.02 ± 0.03	0.02 ± 0.01	0.04 ± 0.06	0.316
PLT (×10^3^/μL)	243 ± 61	249 ± 62	212 ± 44	0.004
MPV (%)	10.8 ± 0.9	10.8 ± 1.0	10.8 ± 0.9	0.984

**Table 4:** Descriptive statistics of BIA parameters in groups of people with different nutritional status assessments.

Bioimpedance Parameters *Me* [Q1; Q3]	MNA		*p*-Value
	Risk of Malnutrition *n* = 29	Proper Nutritional Status *n* = 97	
BMI (kg/m^2^)	25.8 [22.2; 28.4]	27.1 [24.8; 29.8]	0.064
Relative fat mass (%)	41.8 [37.4; 43.4]	40.2 [34.7; 44.7]	0.467
Absolute fat mass (kg)	26.6 [21.4; 30.4]	28.1 [23.8; 33.3]	0.138
Fat-free mass (kg)	38.3 [32.8; 42.1]	42.3 [37.4; 47.3]	0.001
Skeletal muscle mass (kg)	15.8 [12.9; 18.1]	18.7 [16.3; 21.6]	<0.001
SMM TO (kg)	7.06 [5.37; 8.17]	8.16 [7.08; 9.67]	0.001
SMM RL (kg)	3.60 [2.99; 4.02]	4.15 [3.59; 4.91]	<0.001
SMM LL (kg)	3.50 [2.90; 4.02]	4.19 [3.56; 4.81]	<0.001
SMM LA (kg)	0.77 [0.67; 1.00]	0.95 [0.81; 1.16]	0.001
SMM RA (kg)	0.82 [0.72; 0.97]	1.01 [0.88; 1.22]	<0.001
Total Body Water (L)	29.0 [24.8; 31.7]	32.0 [28.4; 35.6]	0.001
Extracellular Water (L)	14.3 [12.1; 15.6]	15.4 [13.7; 17.3]	0.004
R (Ω), 5 kHz, LA	403 [385; 442]	385 [347; 406]	0.008
R (Ω), 5 kHz, RA	393 [379; 432]	377 [336; 403]	0.013
R (Ω), 5 kHz, LL	275 [247; 299]	267 [238; 285]	0.276
R (Ω), 5 kHz, RL	267 [248; 303]	262 [241; 282]	0.310
R (Ω), 5 kHz, LB	715 [640; 755]	667 [618; 717]	0.049
R (Ω), 5 kHz, RB	697 [649; 730]	664 [611; 717]	0.041
R (Ω), 5 kHz, TO	24.3 [23.2; 26.6]	24.5 [22.6; 26.9]	0.596
R (Ω), 7.5 kHz, LA	398 [381; 438]	381 [344; 402]	0.008
R (Ω), 7.5 kHz, RA	391 [376; 429]	373 [333; 400]	0.012
R (Ω), 7.5 kHz, LL	273 [245; 297]	265 [236; 283]	0.256
R (Ω), 7.5 kHz, RL	265 [247; 300]	260 [239; 280]	0.292
R (Ω), 7.5 kHz, LB	709 [636; 749]	662 [612; 711]	0.047
R (Ω), 7.5 kHz, RB	690 [644; 726]	658 [606; 711]	0.040
R (Ω), 7.5 kHz, TO	24.0 [22.9; 26.3]	24.2 [22.3; 26.7]	0.566
R (Ω), 50 kHz, LA	365 [347; 407]	349 [316; 371]	0.011
R (Ω), 50 kHz, RA	359 [346; 399]	343 [307; 370]	0.007
R (Ω), 50 kHz, LL	254 [227; 274]	239 [217; 259]	0.171
R (Ω), 50 kHz, RL	244 [230; 272]	237 [218; 258]	0.135
R (Ω), 50 kHz, LB	649 [594; 697]	606 [551; 649]	0.030
R (Ω), 50 kHz, RB	631 [598; 690]	604 [555; 652]	0.021
R (Ω), 50 kHz, TO	21.5 [20.0; 23.4]	21.2 [19.1; 23.1]	0.360
R (Ω), 75 kHz, LA	357 [337; 398]	341 [309; 363]	0.010
R (Ω), 75 kHz, RA	350 [338; 390]	335 [300; 362]	0.007
R (Ω), 75 kHz, LL	248 [220; 267]	233 [211; 252]	0.147
R (Ω), 75 kHz, RL	237 [225; 265]	231 [213; 250]	0.105
R (Ω), 75 kHz, LB	633 [582; 682]	593 [534; 635]	0.026
R (Ω), 75 kHz, RB	615 [584; 678]	590 [540; 637]	0.018
R (Ω), 75 kHz, TO	20.9 [19.6; 22.8]	20.6 [18.5; 22.4]	0.309
|Xc| (Ω), 5 kHz, LA	12.9 [11.8; 14.4]	13.0 [11.8; 14.1]	0.698
|Xc| (Ω), 5 kHz, RA	13.5 [11.9; 15.2]	13.7 [12.4; 15.0]	0.867
|Xc| (Ω), 5 kHz, LL	8.75 [6.89; 9.93]	9.22 [8.04; 10.62]	0.101
|Xc| (Ω), 5 kHz, RL	9.11 [6.84; 9.68]	9.31 [8.02; 10.54]	0.154
|Xc| (Ω), 5 kHz, LB	22.9 [20.1; 24.9]	23.0 [21.0; 25.6]	0.369
|Xc| (Ω), 5 kHz, RB	23.7 [20.2; 24.8]	24.0 [20.9; 26.3]	0.342
|Xc| (Ω), 5 kHz, TO	1.28 [1.10; 1.47]	1.34 [1.20; 1.56]	0.324
|Xc| (Ω), 7.5 kHz, LA	16.0 [14.0; 17.5]	15.8 [14.3; 17.1]	0.598
|Xc| (Ω), 7.5 kHz, RA	16.4 [14.5; 18.4]	16.4 [14.7; 18.0]	0.963
|Xc| (Ω), 7.5 kHz, LL	10.6 [8.6; 12.2]	11.2 [9.7; 13.0]	0.108
|Xc| (Ω), 7.5 kHz, RL	11.2 [8.2; 11.9]	11.3 [9.8; 13.0]	0.195
|Xc| (Ω), 7.5 kHz, LB	27.2 [24.1; 30.2]	27.8 [24.4; 30.9]	0.396
|Xc| (Ω), 7.5 kHz, RB	28.7 [24.4; 29.7]	28.8 [24.5; 31.4]	0.270
|Xc| (Ω), 7.5 kHz, TO	1.22 [1.12; 1.44]	1.36 [1.20; 1.56]	0.060
|Xc| (Ω), 50 kHz, LA	28.4 [26.3; 30.1]	27.3 [24.6; 29.5]	0.102
|Xc| (Ω), 50 kHz, RA	28.3 [26.8; 30.7]	28.3 [26.1; 30.2]	0.467
|Xc| (Ω), 50 kHz, LL	19.4 [14.9; 21.5]	19.7 [17.0; 22.0]	0.434
|Xc| (Ω), 50 kHz, RL	20.0 [15.4; 21.6]	19.5 [17.2; 22.0]	0.560
|Xc| (Ω), 50 kHz, LB	48.8 [42.2; 51.4]	47.6 [43.2; 52.2]	0.958
|Xc| (Ω), 50 kHz, RB	49.4 [42.3; 51.4]	48.7 [44.1; 53.0]	0.783
|Xc| (Ω), 50 kHz, TO	1.88 [1.63; 2.08]	2.12 [1.78; 2.36]	0.025
|Xc| (Ω), 75 kHz, LA	28.4 [27.3; 30.3]	27.4 [25.0; 29.6]	0.046
|Xc| (Ω), 75 kHz, RA	28.9 [27.2; 31.5]	28.5 [26.1; 30.5]	0.309
|Xc| (Ω), 75 kHz, LL	19.2 [14.7; 20.9]	18.8 [16.5; 21.4]	0.543
|Xc| (Ω), 75 kHz, RL	19.7 [15.2; 21.1]	19.2 [16.7; 21.4]	0.741
|Xc| (Ω), 75 kHz, LB	48.0 [41.7; 50.5]	46.9 [42.4; 51.2]	0.774
|Xc| (Ω), 75 kHz, RB	48.4 [42.8; 50.8]	47.7 [43.4; 51.8]	0.993
|Xc| (Ω), 75 kHz, TO	1.82 [1.55; 1.97]	2.00 [1.72; 2.29]	0.030
Waist circumference (m)	0.90 [0.81; 0.98]	0.91 [0.85; 1.01]	0.335
Weight (kg)	65.2 [55.8; 71.7]	71.0 [63.0; 82.1]	0.004
Height (cm)	1.58 [1.55; 1.63]	1.62 [1.56; 1.68]	0.018
TEE (kcal)	2242 [1893; 2333]	2328 [2070; 2628]	0.034
REE (kcal)	1252 [1168; 1310]	1334 [1244; 1507]	0.002
Energy Content (×10^−3^ kcal)	291 [243; 334]	314 [277; 363]	0.071
FFMI (kg/m^2^)	15.4 [13.5; 16.1]	16.2 [15.2; 17.8]	0.003
FMI (kg/m^2^)	11.1 [8.3; 12.0]	10.6 [8.8; 13.2]	0.616
Z(FFMI)	−1.17 [−2.08; −0.21]	−0.53 [−1.14; 0.18]	0.018
Z(FMI)	0.81 [0.07; 1.16]	0.89 [0.32; 1.51]	0.260
BIA vector R (Ω)	641 [596; 696]	605 [553; 648]	0.025
BIA vector |Xc| (Ω)	−48.6 [−51.5; −41.8]	−48.2 [−52.9; −43.6]	0.880
BIA vector Z(R)	0.54 [−0.21; 1.27]	−0.09 [−0.87; 0.63]	0.020
BIA vector Z(|Xc|)	−0.74 [−1.69; −0.44]	−0.87 [−1.77; −0.39]	0.687
Phase Angle (°)	4.36 [3.95; 4.59]	4.44 [4.20; 4.96]	0.016
Visceral Adipose Tissue	1.77 [1.47; 2.42]	2.02 [1.44; 3.01]	0.417
ECW/TBW (%)	49.9 [47.8; 51.9]	48.2 [46.5; 49.5]	0.001

**Table 5:** Correlation coefficient values between selected BIA parameters and laboratory test results.

	TBW	ECW	TBW/ECW	PA	BMI	BIVA Z(R)
Fe (μg/dL)	0.153	0.098	−0.140	0.100	−0.024	0.083
Ca (mmol/L)	−0.002	−0.012	−0.033	0.005	0.017	0.106
Zn (μmol/L)	0.184 *	0.103	−0.330 ***	0.324 ***	0.034	0.037
HCT (%)	0.371 ***	0.271 **	−0.420 ***	0.366 ***	0.061	0.001
CRP (mg/L)	0.268 **	0.306 ***	−0.033	0.062	0.419 ***	−0.274 **
ALB (g/dL)	0.038	−0.061	−0.289 **	0.293 **	−0.106	0.204 *
Vit. D (ng/mL)	−0.227 *	−0.216 *	0.148	−0.111	−0.183 *	0.041
CHOL (mg/dL)	−0.155	−0.179 *	−0.004	−0.048	−0.153	0.160
Leptin (ng/mL)	0.097	0.180 *	0.107	−0.111	0.377 ***	−0.312 ***

Fe: Iron; CRP: C-Reactive Protein; Ca: Calcium; TG: Triglycerides; AST: Aspartate Aminotransferase; ALT: Alanine Aminotransferase; LDL-D: Low-Density Lipoprotein (Direct measurement); HDL: High-Density Lipoprotein; HGB: Hemoglobin; HCT: Hematocrit; RBC: Red Blood Cell count; MCV: Mean Corpuscular Volume; MCH: Mean Corpuscular Hemoglobin; MCHC: Mean Corpuscular Hemoglobin Concentration; RDW-CV: Red Cell Distribution Width (Coefficient of Variation); WBC: White Blood Cell count; LYMPH: Lymphocytes; MONO: Monocytes; NEUT: Neutrophils; EOS: Eosinophils; BASO: Basophils; IG: Immature Granulocytes; PLT: Platelets (Thrombocytes); MPV: Mean Platelet Volume. The asterisks indicate the level of statistical significance for the Pearson correlation coefficients: * *p* < 0.05; ** *p* < 0.01; *** *p* < 0.001.

## Data Availability

The data presented in this study are available on request from the corresponding author due to privacy restrictions. The raw data contain sensitive patient information, however, anonymized datasets will be made available upon request.

## Supplementary Materials

The following supporting information can be downloaded at: https://www.mdpi.com/article/10.3390/nu17243843/s1, Table S1. Raw segmental and multifrequency bioelectrical impedance parameters (resistance and reactance) of the study participants stratified by sex; Table S2. Spearman’s rank correlation coefficients (*r_s_*) between laboratory test results and bioelectrical impedance analysis (BIA) body composition outcomes; Table S3. Spearman’s rank correlation coefficients (*r_s_*) between laboratory test results and bioelectrical impedance analysis (BIA) body composition outcomes; Table S4. Spearman’s rank correlation coefficients (*r_s_*) between laboratory test results and bioelectrical impedance analysis (BIA) body composition outcomes; Table S5. Spearman’s rank correlation coefficients (*r_s_*) between laboratory test results and bioelectrical impedance vector analysis (BIVA) parameters in a group of 126 elderly individuals. Table S6. Canonical factor loadings and redundancies; Table S7. Canonical Analysis Information Set.
