# Supplementary material for: The Relationship Between Bioelectrical Impedance Analysis Parameters and Laboratory Biomarkers in an Elderly Polish Cohort: A Cross-Sectional Study
Source: Nutrients. 2025 Dec 9;17(24):3843. doi: 10.3390/nu17243843 (PMC12736160; doi:10.3390/nu17243843)
Supplement: Supplementary file 1 [file nutrients-17-03843-s001.zip › nutrients-3983048-supplementary.pdf]

Table S1. Raw segmental and multifrequency bioelectrical impedance parameters (resistance and reactance) of the study participants stratified by sex.

R (Ω), 5 kHz, LA	382.8 ± 49.8	392.9 ± 45.2	328.9 ± 36.9	**<0.001**
R (Ω), 5 kHz, RA	378.5 ± 51.7	388.9 ± 47.6	323.4 ± 35.9	**<0.001**
R (Ω), 5 kHz, LL	265.2 ± 42.3	268.8 ± 43.1	245.9 ± 32.8	**0.011**
R (Ω), 5 kHz, RL	265.4 ± 41.2	268.7 ± 42.4	248.0 ± 29.2	**0.011**
R (Ω), 5 kHz, LB	673.8 ± 88.3	687.9 ± 85.3	598.9 ± 63.5	**<0.001**
R (Ω), 5 kHz, RB	669.5 ± 89.1	683.4 ± 87.2	595.6 ± 58.6	**<0.001**
R (Ω), 5 kHz, TO	24.8 ± 3.4	25.1 ± 3.4	23.5 ± 3.0	**0.037**
R (Ω), 7.5 kHz, LA	379.5 ± 49.6	389.7 ± 45.1	325.6 ± 36.4	**<0.001**
R (Ω), 7.5 kHz, RA	375.2 ± 51.5	385.6 ± 47.4	320.1 ± 35.5	**<0.001**
R (Ω), 7.5 kHz, LL	262.9 ± 42.0	266.6 ± 42.7	243.7 ± 32.3	**0.010**
R (Ω), 7.5 kHz, RL	263.2 ± 40.8	266.5 ± 42.0	245.8 ± 28.7	**0.010**
R (Ω), 7.5 kHz, LB	668.1 ± 87.9	682.3 ± 84.9	593.0 ± 62.6	**<0.001**
R (Ω), 7.5 kHz, RB	663.8 ± 88.7	677.7 ± 86.7	589.8 ± 57.8	**<0.001**
R (Ω), 7.5 kHz, TO	24.6 ± 3.4	24.8 ± 3.4	23.2 ± 3.0	**0.035**
R (Ω), 50 kHz, LA	349.9 ± 48.0	360.1 ± 43.3	295.5 ± 33.0	**<0.001**
R (Ω), 50 kHz, RA	344.5 ± 49.6	355.0 ± 45.3	289.3 ± 32.4	**<0.001**
R (Ω), 50 kHz, LL	240.9 ± 38.3	244.5 ± 39.0	221.8 ± 28.3	**0.004**
R (Ω), 50 kHz, RL	241.1 ± 37.3	244.3 ± 38.5	223.8 ± 24.8	**0.004**
R (Ω), 50 kHz, LB	612.9 ± 83.0	627.1 ± 79.8	537.9 ± 55.3	**<0.001**
R (Ω), 50 kHz, RB	607.8 ± 83.9	621.8 ± 81.6	533.6 ± 51.0	**<0.001**
R (Ω), 50 kHz, TO	21.6 ± 3.1	21.8 ± 3.1	20.2 ± 2.5	**0.015**
R (Ω), 75 kHz, LA	342.0 ± 47.4	352.2 ± 42.7	288.0 ± 32.4	**<0.001**
R (Ω), 75 kHz, RA	336.4 ± 49.0	346.8 ± 44.7	281.6 ± 31.7	**<0.001**
R (Ω), 75 kHz, LL	235.1 ± 37.3	238.6 ± 38.0	216.3 ± 27.5	**0.004**
R (Ω), 75 kHz, RL	235.2 ± 36.4	238.4 ± 37.5	218.2 ± 24.1	**0.004**
R (Ω), 75 kHz, LB	598.7 ± 81.5	612.7 ± 78.3	524.3 ± 54.2	**<0.001**
R (Ω), 75 kHz, RB	593.3 ± 82.5	607.1 ± 80.2	519.8 ± 49.8	**<0.001**
R (Ω), 75 kHz, TO	20.9 ± 3.0	21.2 ± 3.0	19.5 ± 2.4	**0.010**
ǀXcǀ (Ω), 5 kHz, LA	12.9 ± 2.0	13.0 ± 1.9	12.8 ± 2.5	0.712
ǀXcǀ (Ω), 5 kHz, RA	13.7 ± 2.5	13.8 ± 2.5	13.1 ± 2.5	0.264
ǀXcǀ (Ω), 5 kHz, LL	9.1 ± 2.2	9.1 ± 2.2	8.9 ± 2.4	0.608
ǀXcǀ (Ω), 5 kHz, RL	9.2 ± 2.2	9.2 ± 2.1	9.0 ± 2.4	0.665
ǀXcǀ (Ω), 5 kHz, LB	22.9 ± 3.8	22.9 ± 3.6	22.6 ± 4.4	0.714
ǀXcǀ (Ω), 5 kHz, RB	23.5 ± 4.2	23.6 ± 4.2	23.0 ± 4.5	0.508
ǀXcǀ (Ω), 5 kHz, TO	1.4 ± 0.4	1.4 ± 0.4	1.4 ± 0.4	0.684
ǀXcǀ (Ω), 7.5 kHz, LA	15.7 ± 2.4	15.7 ± 2.3	15.7 ± 3.0	0.951
ǀXcǀ (Ω), 7.5 kHz, RA	16.4 ± 2.7	16.5 ± 2.7	16.1 ± 3.0	0.602
ǀXcǀ (Ω), 7.5 kHz, LL	11.1 ± 2.8	11.1 ± 2.7	10.9 ± 3.0	0.770
ǀXcǀ (Ω), 7.5 kHz, RL	11.2 ± 2.7	11.3 ± 2.7	11.1 ± 3.0	0.838
ǀXcǀ (Ω), 7.5 kHz, LB	27.3 ± 4.6	27.3 ± 4.5	27.5 ± 5.5	0.852
ǀXcǀ (Ω), 7.5 kHz, RB	28.0 ± 5.0	28.1 ± 4.9	28.0 ± 5.6	0.948
ǀXcǀ (Ω), 7.5 kHz, TO	1.36 ± 0.31	1.34 ± 0.31	1.42 ± 0.30	0.311
ǀXcǀ (Ω), 50 kHz, LA	27.3 ± 3.5	27.6 ± 3.4	25.8 ± 3.6	**0.030**
ǀXcǀ (Ω), 50 kHz, RA	28.2 ± 3.7	28.6 ± 3.6	26.4 ± 3.7	**0.017**
ǀXcǀ (Ω), 50 kHz, LL	19.1 ± 4.4	19.3 ± 4.4	18.2 ± 3.9	0.315
ǀXcǀ (Ω), 50 kHz, RL	19.4 ± 4.3	19.6 ± 4.4	18.5 ± 4.0	0.316
ǀXcǀ (Ω), 50 kHz, LB	47.4 ± 7.3	47.7 ± 7.4	45.4 ± 7.0	0.195

|Xc| (Ω), 50 kHz, RB	48.2 ± 7.4	48.5 ± 7.5	46.2 ± 7.3	0.211
|Xc| (Ω), 50 kHz, TO	2.04 ± 0.43	2.00 ± 0.41	2.27 ± 0.44	**0.010**
|Xc| (Ω), 75 kHz, LA	27.7 ± 3.6	28.1 ± 3.5	25.5 ± 3.3	**0.002**
|Xc| (Ω), 75 kHz, RA	28.6 ± 3.8	29.0 ± 3.6	26.1 ± 3.4	**0.001**
|Xc| (Ω), 75 kHz, LL	18.7 ± 4.2	18.9 ± 4.2	17.5 ± 3.6	0.191
|Xc| (Ω), 75 kHz, RL	19.0 ± 4.1	19.2 ± 4.2	17.8 ± 3.6	0.184
|Xc| (Ω), 75 kHz, LB	46.6 ± 7.1	47.1 ± 7.1	44.0 ± 6.3	0.069
|Xc| (Ω), 75 kHz, RB	47.4 ± 7.2	47.8 ± 7.2	44.8 ± 6.6	0.083
|Xc| (Ω), 75 kHz, TO	1.94 ± 0.46	1.89 ± 0.44	2.20 ± 0.43	**0.004**
BIA vector R (Ω)	610 ± 83	624 ± 80	536 ± 52	**<0.001**
BIA vector |Xc| (Ω)	-47.8 ± 7.3	-48.1 ± 7.3	-45.8 ± 7.1	0.197
BIA vector Z(R)	0.05 ± 1.31	0.06 ± 1.38	0.01 ± 0.84	0.875
BIA vector Z(|Xc|)	-0.98 ± 1.02	-0.96 ± 1.06	-1.13 ± 0.85	0.494

Table S2. Spearman's rank correlation coefficients (*rs*) between laboratory test results and bioelectrical impedance analysis (BIA) body composition outcomes.

Laboratory test results	BMI (kg/m²)	RFM (kg)	AFM (kg)	FFM (kg)	SMM (kg)	TBW (L)	ECW (L)
Ghrelin (ng/ml)	0.139	0.159	0.158	0.008	0.014	0.010	0.014
Leptin (ng/ml)	0.660	0.666	0.727	0.131	0.132	0.134	0.180
Fe (µg/dl)	-0.097	-0.088	-0.060	0.107	0.123	0.109	0.089
CRP (mg/l)	0.429	0.333	0.439	0.204	0.222	0.224	0.229
Ca (mmol.l)	-0.014	0.028	0.040	0.011	0.020	0.002	-0.031
Albumins (g/dl)	-0.092	-0.063	-0.048	0.034	0.063	0.026	-0.052
Zn (µmol/l)	-0.025	-0.190	-0.048	0.175	0.223	0.174	0.084
Vitamin D (ng/ml)	-0.158	-0.008	-0.135	-0.202	-0.206	-0.203	-0.182
Total cholesterol (mg/dl)	-0.159	-0.034	-0.098	-0.124	-0.095	-0.119	-0.164
HDL (mg/dl)	-0.344	0.026	-0.214	-0.395	-0.387	-0.396	-0.385
Non-cholesterol (mg/dl)	-0.054	-0.031	-0.033	-0.004	0.022	0.002	-0.047
LDL-D (mg/dl)	-0.116	-0.048	-0.077	-0.065	-0.042	-0.058	-0.098
TG (mg/dl)	0.298	0.116	0.228	0.233	0.261	0.228	0.176
AST (IU/l)	-0.039	-0.116	-0.088	0.093	0.064	0.087	0.105
ALT (IU/l)	0.238	0.072	0.168	0.220	0.221	0.211	0.176
HGB (g/dl)	0.045	-0.191	0.051	0.400	0.432	0.396	0.307
HCT (%)	0.046	-0.148	0.065	0.359	0.391	0.358	0.282
RCB (×10⁶/µl)	0.076	-0.125	0.087	0.348	0.384	0.351	0.277
MCV (fl)	-0.077	0.008	-0.050	-0.044	-0.047	-0.049	-0.045
MCH (pg)	-0.109	-0.097	-0.084	0.065	0.072	0.055	0.022
MCHC (g/dl)	-0.099	-0.207	-0.091	0.206	0.228	0.198	0.123
RDW-CV (%)	0.092	0.081	0.056	-0.011	-0.030	-0.008	0.032
WBC (×10³/µl)	0.250	0.084	0.228	0.157	0.158	0.152	0.134
LYMPH (×10³/µl)	0.233	0.086	0.206	0.145	0.155	0.138	0.097
MONO (×10³/µl)	0.196	-0.031	0.160	0.252	0.224	0.249	0.267
NEUT (×10³/µl)	0.166	0.046	0.152	0.102	0.100	0.099	0.091
EOS (×10³/µl)	0.126	0.043	0.029	0.056	0.031	0.061	0.087
BASO (×10³/µl)	0.116	0.065	0.126	0.074	0.060	0.075	0.083
IG (×10³/µl)	0.186	0.083	0.196	0.143	0.161	0.140	0.109

PLT (×10³/µl)	0.170	0.186	0.181	-0.061	-0.012	-0.058	-0.103
MPV (%)	-0.035	0.109	0.002	-0.093	-0.110	-0.092	-0.058

BMI - Body Mass Index, RFM - Relative Fat Mass, AFM - Adipose Tissue Mass, SMM - Skeletal Muscle Mass, TBW - Total Body Water, ECW - Extracellular Water

Table S3. Spearman's rank correlation coefficients (rS) between laboratory test results and bioelectrical impedance analysis (BIA) body composition outcomes.

Laboratory test results	WC (cm)	Weight (kg)	Hight (cm)	TEE (kcal/d)	REE (kcal/d)	EC (kcal)
Ghrelin (ng/ml)	0.081	0.113	-0.033	0.077	0.075	0.156
Leptin (ng/ml)	0.385	0.412	-0.156	0.136	0.225	0.688
Fe (µg/dl)	0.045	0.064	0.146	0.219	0.141	-0.044
CRP (mg/l)	0.337	0.384	-0.003	0.262	0.296	0.437
Ca (mmol.l)	0.029	0.035	0.094	0.005	0.013	0.048
Albumins (g/dl)	-0.045	0.020	0.180	-0.024	0.039	-0.024
Zinc (µmol/l)	0.077	0.096	0.194	0.166	0.174	-0.023
Vitamin D (ng/ml)	-0.042	-0.189	-0.088	-0.096	-0.185	-0.151
Total cholesterol (mg/dl)	-0.159	-0.119	0.007	-0.126	-0.054	-0.110
HDL (mg/dl)	-0.328	-0.395	-0.148	-0.344	-0.372	-0.256
Non-cholesterol (mg/dl)	-0.058	0.004	0.043	-0.026	0.069	-0.033
LDL-D (mg/dl)	-0.119	-0.063	0.039	-0.084	0.001	-0.081
TG (mg/dl)	0.264	0.301	-0.011	0.223	0.303	0.244
AST (IU/l)	-0.092	0.029	0.087	0.024	0.040	-0.069
ALT (IU/l)	0.119	0.242	0.062	0.245	0.234	0.186
HGB (g/dl)	0.178	0.306	0.418	0.377	0.408	0.105
HCT (%)	0.159	0.286	0.375	0.319	0.369	0.113
RCB (×10⁶/µl)	0.111	0.290	0.361	0.293	0.357	0.132
MCV (fl)	0.061	-0.043	-0.049	0.004	-0.024	-0.051
MCH (pg)	0.083	0.014	0.101	0.122	0.085	-0.073
MCHC (g/dl)	0.048	0.095	0.287	0.242	0.200	-0.067
RDW-CV (%)	0.042	0.014	-0.117	-0.085	-0.018	0.045
WBC (×10³/µl)	0.309	0.239	-0.003	0.235	0.217	0.257
LYMPH (×10³/µl)	0.191	0.211	-0.034	0.125	0.161	0.227
MONO (×10³/µl)	0.305	0.280	0.192	0.297	0.286	0.200
NEUT (×10³/µl)	0.260	0.156	0.007	0.186	0.153	0.174
EOS (×10³/µl)	0.118	0.069	-0.112	-0.010	0.002	0.040
BASO (×10³/µl)	0.196	0.123	0.009	0.044	0.061	0.137
IG (×10³/µl)	0.276	0.199	0.079	0.209	0.206	0.212
PLT (×10³/µl)	-0.012	0.049	-0.134	0.001	-0.012	0.161
MPV (%)	-0.043	-0.047	-0.007	-0.058	-0.030	-0.005

WC - waist circumference, Weight – body mass, Hight – body hight, TEE - total energy expenditure, REE - resting energy expenditure, EC - energy content,

Table S4. Spearman's rank correlation coefficients (r_S) between laboratory test results and bioelectrical impedance analysis (BIA) body composition outcomes.

Laboratory test results	FFMI (kg/m²)	FMI (kg/m²)	Z(FFMI) (-)	Z(FMI) (-)	VAT (L)	ECW/TBW (-)
Ghrelin (ng/ml)	0.023	0.152	0.006	0.166	0.073	0.061
Leptin (ng/ml)	0.246	0.763	0.501	0.692	0.222	0.136
Fe (µg/dl)	0.057	-0.107	-0.064	-0.080	0.113	-0.114
CRP (mg/l)	0.261	0.426	0.305	0.429	0.281	-0.030
Ca (mmol.l)	-0.049	0.009	-0.061	0.014	0.068	-0.047
Albumins (g/dl)	-0.061	-0.106	-0.133	-0.056	0.063	-0.218
Zinc (µmol/l)	0.139	-0.131	-0.042	-0.030	0.173	-0.340
Vitamin D (ng/ml)	-0.218	-0.087	-0.140	-0.130	-0.048	0.141
Total cholesterol (mg/dl)	-0.170	-0.093	-0.145	-0.136	-0.141	-0.041
HDL (mg/dl)	-0.449	-0.149	-0.286	-0.271	-0.323	0.211
Non-cholesterol (mg/dl)	-0.030	-0.044	-0.066	-0.051	-0.034	-0.098
LDL-D (mg/dl)	-0.108	-0.083	-0.107	-0.107	-0.101	-0.056
TG (mg/dl)	0.322	0.208	0.202	0.288	0.283	-0.186
AST (IU/l)	0.098	-0.115	0.030	-0.068	-0.086	-0.040
ALT (IU/l)	0.282	0.143	0.244	0.203	0.115	-0.246
HGB (g/dl)	0.257	-0.106	0.006	0.042	0.279	-0.465
HCT (%)	0.222	-0.080	0.008	0.045	0.247	-0.392
RCB (×10⁶/µl)	0.214	-0.045	0.054	0.070	0.185	-0.379
MCV (fl)	-0.036	-0.048	-0.118	-0.050	0.087	0.037
MCH (pg)	0.033	-0.123	-0.160	-0.068	0.158	-0.140
MCHC (g/dl)	0.113	-0.173	-0.101	-0.079	0.144	-0.357
RDW-CV (%)	0.023	0.111	0.053	0.086	-0.003	0.158
WBC (×10³/µl)	0.223	0.183	0.104	0.271	0.327	-0.093
LYMPH (×10³/µl)	0.207	0.185	0.125	0.246	0.191	-0.121
MONO (×10³/µl)	0.260	0.066	0.081	0.189	0.335	-0.077
NEUT (×10³/µl)	0.155	0.115	0.074	0.177	0.270	-0.071
EOS (×10³/µl)	0.144	0.073	0.150	0.063	0.082	0.051
BASO (×10³/µl)	0.094	0.120	0.073	0.133	0.170	0.004
IG (×10³/µl)	0.159	0.139	0.071	0.208	0.327	-0.111
PLT (×10³/µl)	0.006	0.214	0.083	0.188	-0.029	-0.074
MPV (%)	-0.110	0.029	-0.077	-0.001	-0.012	0.144

FFMI - fat-free mass index, FMI - fat mass index, Z(FFMI) - Z-score for FFMI,

Z(FMI) - Z-score for FMI, VAT - visceral adipose tissue, ECW/TBW - extracellular water by total body water

Table S5. Spearman's rank correlation coefficients (*rs*) between laboratory test results and bioelectrical impedance vector analysis (BIVA) parameters in a group of 126 elderly individuals.

Laboratory test results	BIVA R (Ω)	BIVA Xc (Ω)	BIVA Z(R)	BIVA Z(Xc)	PA (°)
Ghrelin (ng/ml)	0.025	0.013	0.058	-0.003	-0.022
Leptin (ng/ml)	-0.148	0.103	-0.313	-0.130	0.015
Fe (µg/dl)	-0.040	-0.038	0.020	0.025	0.101
CRP (mg/l)	-0.200	0.125	-0.239	-0.152	0.052
Ca (mmol.l)	0.157	-0.208	0.167	0.179	0.039
Albumins (g/dl)	0.124	-0.361	0.152	0.321	0.271
Zn (µmol/l)	-0.050	-0.250	0.072	0.241	0.321
Vitamin D (ng/ml)	0.102	0.011	0.050	-0.012	-0.116
Total cholesterol (mg/dl)	0.225	-0.161	0.173	0.125	-0.006
HDL (mg/dl)	0.414	-0.151	0.242	0.110	-0.204
Non-cholesterol (mg/dl)	0.107	-0.115	0.111	0.093	0.054
LDL-D (mg/dl)	0.170	-0.117	0.140	0.088	-0.001
TG (mg/dl)	-0.229	-0.032	-0.096	0.062	0.238
AST (IU/l)	-0.114	0.063	-0.050	-0.050	-0.011
ALT (IU/l)	-0.205	-0.091	-0.158	0.087	0.277
HGB (g/dl)	-0.132	-0.191	-0.012	0.142	0.360
HCT (%)	-0.134	-0.138	-0.038	0.089	0.317
RCB ($\times 10^6$/µl)	-0.131	-0.115	-0.079	0.053	0.314
MCV (fl)	0.011	-0.005	0.094	0.034	-0.060
MCH (pg)	-0.010	-0.108	0.124	0.120	0.063
MCHC (g/dl)	-0.050	-0.171	0.048	0.138	0.212
RDW-CV (%)	-0.041	0.145	-0.025	-0.088	-0.075
WBC ($\times 10^3$/µl)	-0.174	0.024	-0.038	0.016	0.129
LYMPH ($\times 10^3$/µl)	-0.131	-0.080	-0.038	0.118	0.180
MONO ($\times 10^3$/µl)	-0.231	0.158	-0.077	-0.132	0.008
NEUT ($\times 10^3$/µl)	-0.124	0.037	-0.033	-0.015	0.089
EOS ($\times 10^3$/µl)	-0.196	0.103	-0.183	-0.068	0.037
BASO ($\times 10^3$/µl)	-0.087	0.021	-0.039	0.001	0.019
IG ($\times 10^3$/µl)	-0.087	0.015	-0.043	-0.010	0.099
PLT ($\times 10^3$/µl)	0.017	-0.131	-0.011	0.141	0.130
MPV (%)	0.100	0.040	0.055	-0.050	-0.128

Coefficients significant at $p < 0.05$ were highlighted, BIVA R – resistance in Bioelectrical Impedance Vector Analysis, BIVA Xc – reactance in Bioelectrical Impedance Vector Analysis,

BIVA Z(R) - Z-score for resistance (R), BIVA Z(Xc) - Z-score for reactance (Xc), PA – phase angle

Table S6. Canonical factor loadings and redundancies.

LEPTIN^{trans}	**0,978**	-0,179	0,027	0,038	0,075	0,010	-0,065
Fe	-0,043	**0,518**	0,016	-0,544	0,097	0,065	0,648
Zn^{trans}	-0,151	**0,649**	0,183	0,267	0,261	0,603	-0,139
Vit D	-0,184	-0,407	-0,252	-0,155	0,693	0,424	0,231
CHOL	-0,041	-0,282	0,764	-0,273	-0,300	0,255	0,324
RDW	0,107	-0,186	-0,362	0,690	-0,392	0,053	0,435
MPV	-0,022	0,107	-0,382	-0,478	-0,589	0,503	-0,120

Extracted variance (proportions), LAB set		
Canonical Factor	Extracted Variance	Redundancy
Factor 1	0.147	**0.094**
Factor 2	0.145	0.031
Factor 3	0.137	0.021
Factor 4	0.167	0.020
Factor 5	0.165	0.012
Factor 6	0.124	0.005
Factor 7	0.115	0.000

BMI	**0,835**	0,280	-0,200	0,166	0,085	-0,237	-0,305
RFM	**0,829**	-0,311	-0,165	-0,166	-0,133	0,272	-0,189
ECW	0,308	0,583	-0,111	0,116	-0,088	-0,669	-0,061
WC	0,648	0,409	-0,185	0,027	0,168	-0,062	-0,167
TEE	0,361	**0,710**	-0,050	-0,241	0,322	-0,433	-0,023
BIVA R	-0,305	-0,263	0,323	-0,344	-0,278	0,695	0,059
BIVA Xc	0,145	-0,235	-0,483	0,136	-0,058	-0,765	-0,133
Phase Angle (°)	0,116	**0,628**	0,207	0,179	0,323	0,233	0,022
ECW/TBW	0,036	**-0,667**	-0,529	0,044	-0,257	0,105	0,018

Extracted variance (proportions), right set		
Canonical Variable	Extracted Variance	Redundancy
Variable 1	0.240	0.154
Variable 2	0.239	0.052
Variable 3	0.086	0.013
Variable 4	0.033	0.004
Variable 5	0.046	0.003
Variable 6	0.212	0.009
Variable 7	0.020	0.000

Table S7. Canonical Analysis Information Set.

	LAB set	BIA set
N=113		
Number of variables	7	9
Extracted variance	100.000%	87.6188%
Total redundancy	18.3688%	23.5316%
Variables:		
	LEPTIN	BMI
	Fe	RFM
	Zn	ECW
	Vit D	WC
	CHOL	TEE
	RDW	BIVA R
	MPV	BIVA Xc
		Phase Angle (°)
		ECW/TBW
